# The role of socioeconomic status and political attitudes in the spread of Covid-19 in Austria

**DOI:** 10.1093/eurpub/ckac130.226

**Published:** 2022-10-25

**Authors:** A Stradner

**Affiliations:** 1 International Affairs and Consulting, The Austrian National Public Health Institute, Vienna, Austria; 2 Socioeconomics Department, Vienna University of Economics and Business, Vienna, Austria

## Abstract

This study investigates the role of socioeconomic status (SES) and political attitudes marked by populism and libertarianism in the spread of Covid-19 infections across Austria. A spatial regression approach is adopted based on official registry data on Covid-19 cases at the municipality level, granted by the Austrian National Public Health Institute. This allows the consideration of spatial dependencies between observations in close geographic proximity. Moreover, to uncover potential temporal (in)stabilities in the effects, the associations are examined over two pandemic phases, namely the second (06/20 to 02/21) and third wave (02/21 to 07/21) of infections. The analysis shows that low educational attainment and income led to higher infection rates during both periods under investigation. In contrast, unemployment was negatively related to the Covid-19 incidence. While the findings for income and education were more pronounced during the third wave of infections, unemployment had a more significant impact during the second wave. Contrary to what was expected, the findings report that populist attitudes were associated with lower case numbers during the second wave. This association reversed and became positive in the third wave but was no longer significant. A positive yet non-significant relationship was detected between libertarian attitudes and Covid-19 cases for both periods studied. The findings suggest that low-income and less-educated groups carry a higher Covid-19 disease burden in Austria. This provides vital information for policymakers to develop targeted public health strategies to protect vulnerable groups and achieve a more equitable distribution of health in society. Only limited evidence was provided regarding the impact of populist and libertarian sentiments on the spread of Covid-19. As polarisation has increased throughout the pandemic, further research on attitudes characterized by skepticism towards state intervention and science is needed.

**Key messages:**

- This study shows that income, education, and unemployment had a significant impact on municipality-level Covid-19 infection rates in Austria during the second and third waves of the pandemic.
- While populist and libertarian attitudes were positively associated with the Covid-19 incidence during the third wave, the effects were not significant.

